# The prevalence of constant supportive observations in high, medium and low secure services

**DOI:** 10.1192/bjb.2017.14

**Published:** 2018-04

**Authors:** Katie Lambert, Simon Chu, Chris Duffy, Victoria Hartley, Alison Baker, Jane L. Ireland

**Affiliations:** 1 Ashworth Research Centre, Mersey Care NHS Foundation Trust, Maghull; 2 University of Central Lancashire, Preston; 3 Ashworth Hospital, Mersey Care NHS Foundation Trust, Maghull

## Abstract

**Aims and method:**

We explored the prevalence and use of constant supportive observations (CSO) in high, medium and low secure in-patient services in a single National Health Service (NHS) mental health trust. From clinical records, we extracted data on the length of time of CSO, the reason for the initiation of CSO and associated adverse incidents for all individuals who were placed on CSO between July 2013 and June 2014.

**Results:**

A small number of individuals accounted for a disproportionately large proportion of CSO hours in each setting. Adverse incident rates were higher on CSO than when not on CSO. There was considerable variation between different settings in terms of CSO use and the reasons for commencing CSO.

**Clinical implications:**

The study describes the prevalence and nature of CSO in secure forensic mental health services and the associated organisational costs. The marked variation in CSO use between settings suggests that mental health services continue to face challenges in balancing risk management with minimising restrictive interventions.

**Declaration of interest:**

A.B. and J.L.I. are both directly employed by the NHS trust in which the study was conducted.

Supportive observations involve the increased monitoring of mental health in-patients who are at an increased risk of harming themselves, harming others or absconding.^1^ The practice involves clinical staff being assigned to an unsettled patient to engage with them therapeutically^2^ and monitor their well-being.^3^ The National Institute for Health and Care Excellence (NICE) guidance on the short-term management of violent behaviour in in-patient settings^4^ outlines four distinct levels of observations, with the highest two levels (3 and 4) involving constant observation of the patient. Observations may also vary in terms of the number of staff assigned to conduct them – constant supportive observations (CSO) are commonly conducted on a one-to-one basis, but in more extreme cases multiple staff may be assigned to observations of the same patient (the most recent NICE guideline on the management of aggression has formalised observations involving multiple staff as a distinct category^5^).

Observations are the recommended approach for managing individuals deemed to be at risk,^6^ reflecting the competing demands of a mental health service – the provision of compassionate care while managing risk – but the rationale and efficacy of CSO is debated in the literature.^7^^–^^9^ However, there are no reports of the prevalence of supportive observations in mental health services, and so the extent to which supportive observations are being utilised in mental health services, and in what context, is unclear. This research therefore explored the prevalence and nature of CSO in a single National Health Service (NHS) mental health trust, comparing data from high, medium and low secure forensic mental health services within that trust.

## Method

The data were gathered from a single NHS mental health trust in England for the 12-month period between 1 July 2013 and 30 June 2014. The secure division within this trust comprises a high secure service (HSS; 228 male in-patient beds), a medium secure unit (MSU; 58 male in-patient beds and eight female in-patient beds) and a low secure unit (LSU; 32 male in-patient beds). During the period of study, the mean daily occupancy levels were at 84% of capacity for high secure, 88% for male medium secure, 93% for female medium secure and 90% for low secure (not including beds assigned to patients on trial leave).

From clinical records, we identified all individuals who were resident on 1 July 2013 or admitted between 1 July 2013 and 30 June 2014 in each service. The clinical information for each individual was manually searched, and we extracted and collated data on all episodes of level 3 or 4 observations that took place within the review period. For each episode, the data recorded were: the level of observations (level 3 or 4), the start and end time/date of each episode of observation, the number of staff involved in each episode, and the reason for initiation of the episode. An episode of CSO was only recorded when there was a defined start and stop time recorded in clinical notes. Thus, where the CSO arrangement was flexible dependent on circumstances (e.g. level 2 in communal areas but level 3 when in their bedroom) and no start/stop time was recorded in the notes for the changing levels of CSO, it was not possible to record the data for those episodes. Data on all adverse incidents involving the individuals on CSO were collated and manually searched to establish the number of incidents that occurred while that individual was on CSO and not on CSO.

## Results

During the review period, 239 individuals were resident in the HSS at different periods and 56 of these were placed on CSO at some stage during the year. In the female MSU ward, nine different individuals were resident during the year and eight of these were placed on CSO. There were 84 individual residents during the year on the male MSU wards, with 31 of these being placed on CSO. Eighteen of the 38 residents in the LSU during the year were placed on CSO. There were only a very small number of episodes of level 4 observations across the data-set (seven episodes in HSS, two episodes in MSU, and four episodes in LSU) and so for the purposes of analysis, these were combined with the level 3 episodes. A summary of the data extracted for each unit is shown in Table 1.

**Table 1:** The prevalence of constant supportive observations in low, medium and high secure services between July 2013 and June 2014

		Medium secure		
	Low secure	Male wards	Female ward	High secure
OBD for the period	10 454	18 626	2726	69 776
No. of patients on CSO (no. of patients resident during the period)	18 (38)	31 (84)	8 (9)	56 (239)
Total patient hours spent on CSO	14 960	15 770	14 025	41 192
Total staff hours spent on CSO	16 537	18 662	16 468	47 628
Patient hours per 100 OBD	143.10	84.67	514.49	59.03
Staff hours per 100 OBD	158.18	100.19	604.11	68.26
Estimated cost per 100 OBD, £ (estimated total cost for unit, £)^a^	3734 (390 370)	1811 (337 269)	10 802 (294 471)	1221 (851 636)
Reasons for initiating CSO, % staff hours	Aggression 47 Self-harm 14 Deterioration 0 Env. change 2 Other 37	Aggression 45 Self-harm 34 Deterioration 6 Env. change 14 Other 1	Aggression 8 Self-harm 88 Deterioration 1 Env. change 3 Other 0	Aggression 8 Self-harm 52 Deterioration 18 Env. change 21 Other

OBD, occupied bed days; CSO, constant supportive observations; Env. change, environment change.

a. Estimates based on one-third of CSO conducted by staff in band 5, two-thirds conducted by staff in band 3.

### Prevalence of constant observations

Across the secure division, patients were placed on CSO for a total of 85 947 h in the 12-month period of study. Table 1 shows the prevalence of CSO in the three different units, both in terms of the number of hours that patients spent on CSO and the number of staff hours devoted to CSO. To allow comparison between units, occupied bed days (OBD; the sum of the daily number of occupied beds for the entire period) was used as the common denominator. Table 1 shows the total number of hours of CSO per 100 OBD in each unit. The HSS recorded the lowest CSO levels (59 h per 100 OBD), while the highest level of CSO was in the female MSU (514 h per 100 OBD). Staff hours devoted to CSO were consistently higher than patient hours, owing to episodes of 2:1 or 3:1 observation, and the magnitude of increase in staff hours varied between 10 and 18% of patient hours (low secure: 10% higher; male medium secure: 18% higher; female medium secure: 17% higher; high secure: 15% higher).

### Distribution of CSO hours across patients

CSO was not evenly distributed across the in-patient population; in all units, a small number of individuals accounted for a substantial proportion of staff time on CSO. In the HSS, five out of 56 individuals on CSO accounted for 54% of all CSO hours in the unit (one individual was on CSO for the entire 12-month period). On the female MSU ward, two out of eight individuals on CSO accounted for 57% of the CSO hours, while on the male MSU wards, four out of 31 individuals on CSO accounted for 56% of the CSO hours. Similarly, in the LSU, two out of 18 individuals accounted for 46% of the CSO hours.

### Reason for commencing CSO

To aggregate the reasons for commencing CSO, the circumstance of each episode of CSO was extracted from clinical notes and placed in one of five categories. Reasons were categorised as ‘Aggression’ if the individual was placed on CSO because of an act of aggression (including violence) or threats of aggression, including verbal abuse. ‘Self-harm’ included actual self-harm as well as threatened self-harm and requests from the individual for extra support because they feared harming themselves in the near future. CSO was categorised as ‘Deterioration’ when individuals were placed on CSO because of a general agitation or deterioration in mental state that warranted additional support for them. CSO was categorised as ‘Environment Change’ when a specific change in environment preceded CSO and was the main reason for it, e.g. new admission, termination of a period of seclusion, ward change. CSO was categorised as ‘Other’ when there was a specific reason for placing an individual on CSO that did not fit the other categories, e.g. for safeguarding purposes in a situation where there was a high likelihood of exploitation, or for physical health purposes when an individual had recently returned from hospital after an operation and required support.

The proportion of staff hours devoted to CSO in each of the different categories in each unit is shown in Table 1, where it can be seen that the reasons for initiation of CSO varied between services. In HSS and the female MSU, self-harm (actual or risk of) was the main reason for CSO, while aggression was not often a reason to initiate CSO. In both the male MSU and the LSU, however, aggression was the predominant reason for initiating CSO. CSO to provide support in self-harm was also a frequent motive in the male MSU, while a large proportion of CSO hours were devoted to a specific other reason in the LSU. Further exploration of the LSU reasons indicated that a single individual was placed on CSO for a 6-month period for safeguarding reasons, leading to the unusually high proportion of CSO hours in the ‘other’ category.

### Adverse incidents

To examine the effect of CSO on adverse incidents, we extracted data on the date/time of each incident and the incident category for every incident involving any individual on CSO during the period of study. By examining the date/time and circumstances of the incident, it was possible to categorise each incident as having occurred while the individual was either on CSO or not on CSO. Using data on the number of hours that all individuals spent on CSO within a unit, and also the number of hours for those same individuals during the 12-month period that were not spent on CSO, we computed the incident rates relative to a common denominator (per 100 h on CSO and not on CSO) to enable comparisons between incident rates.

Incidents are categorised in terms of nature and severity into four categories, A–D. Category A incidents are very serious incidents that may involve a death, serious sexual assault or hostage-taking. No individuals on CSO during the period of study were involved in any category A incidents. Examples of category B incidents include attempted hostage-taking, serious assault and attempted suicide; typical category C incidents may involve assault, moderate self-harm and threats; and examples of Category D incidents may include verbal abuse, minor self-harm and property damage. Table 2 shows the number of adverse incidents (in each category) per 100 h of residence while those individuals were on CSO and not on CSO.

**Table 2:** Adverse incidents in each category and in total for each unit for patients involved in constant supportive observations (CSO)

	Incidents while on CSO^a^				Incidents while not on CSO^a^			
	Category				Category			
Unit	B	C	D	Total	B	C	D	Total
Low secure	0.01	0.05	0.35	0.41	0.01	0.02	0.16	0.20
Medium secure – male	0.02	0.08	0.18	0.27	0.01	0.03	0.07	0.10
Medium secure – Female	0.01	0.07	0.53	0.61	0.00	0.04	0.12	0.16
High secure	0.0	0.43	0.62	1.06	0.01	0.12	0.22	0.35
High secure (without atypical patient)^b^	0.01	0.21	0.36	0.58				

a. Incidents are presented per 100 h of residence.

b. Atypical patient was on CSO for the entire 12-month period. Category B: serious incident (e.g. attempted hostage taking, serious assault, attempted suicide); category C: less serious incident (e.g. assault, moderate self-harm, threats); category D: minor incident (e.g. verbal abuse, minor self-harm, property damage).

In all units, figures indicate that adverse incidents were more common for individuals on CSO than when those same individuals were not on CSO, with the highest overall incident rate for patients on CSO in the HSS (1.06 incidents per 100 h). Further examination of the records from HSS revealed that one individual was responsible for 251 incidents while on observations; because he was on CSO for the entire 12-month period, there were no instances of incidents while not on observations. When this atypical individual was removed from the incident figures, the incident rate for HSS patients while on CSO reduced to 0.58 incidents per 100 h.

The data in Table 2 indicate that across all units, individuals were at least twice as likely to engage in an incident while on CSO compared with when they were not on CSO, and, in the case of the female MSU ward, almost four times as likely. Closer examination of the figures revealed that this was driven largely by an increase in category D incidents when on CSO.

### Organisational costs of observations

The exact cost to the organisation of conducting CSO depends on the hourly rate of pay for the staff that conduct the observations. The identity of the staff who contributed to conducting each episode of CSO was not available in the clinical notes, and so the true costs could not be calculated. However, we estimated staff costs by using the 2013/2014 hourly rates for NHS band 3 (£16.07) and band 5 (£21.51), based on the informal estimate from hospital managers that one-third of CSO was carried out by clinical staff in band 5 and two-thirds by clinical staff in band 3. On this basis, we estimated the cost of CSO to be £851 636 for the HSS, £631 740 for the MSU and £390 370 for the LSU, noting that the female ward accounted for 47% of the total cost of CSO in the MSU (£294 471 for the female ward). When OBD is used as a common denominator, the relative costs of CSO in each service may be compared; the estimated figures in Table 1 show that the cost of CSO in the HSS was £1221 per 100 OBD, compared with £10 802 in the female MSU.

## Discussion

This paper represents the first published study of the prevalence of supportive observations in a UK mental health trust, reporting data from low, medium and high secure services.

In the literature, the organisational cost of observations is reported to be high. One economic analysis in 2008 estimated the annual cost of observations to the NHS at £80 m^10^ with £35 m spent on providing CSO. In the present data-set, our findings estimate that the cost to the organisation of constant observations alone was £1.8 m in 2013–14. Estimates suggest that between 3 and 20% of people admitted into mental health services will be subject to some form of intensive observation, and that up to 20% of the nursing budget for a hospital may be used in the provision of constant observations.^7^ In the current economic climate, where cost, value and effectiveness are increasingly important, it is appropriate to consider the use of finite staffing resources. Although constant observations remain fundamental to mental health nursing care, some researchers question the efficacy of the practice,^8^ particularly against the more contemporary background of reducing restrictive practices in mental healthcare, and clinical decisions on the issue of CSO remain a policy matter for individual mental health trusts.

A small number of individuals in each unit accounted for a disproportionately large amount of the time devoted to conducting CSO and thus a large proportion of the CSO costs to the organisation. In the MSU, for example, two patients out of 93 individuals who were resident on that unit accounted for 27% of the total CSO hours in the whole unit. Similarly, in the HSS, two patients out of the 239 individuals who were resident that year accounted for 31% of the CSO hours in that unit. In secure mental health services, it is not unusual for some patients to have high dependency needs, and assessing the balance between pro-active and defensive approaches to managing risk can be a challenge. As a result, some patients are subjected to constant observations for sustained periods of time; as all mental health trusts are motivated to employ the least restrictive interventions while maintaining safety, this is a difficult balance to strike, with some trusts potentially erring on the side of caution.

The finding that patients were involved in adverse incidents more frequently when on observations than when not on observations could reflect closer surveillance of behaviour, but is more likely explained by different baseline presentations in the two contexts. Individuals are more disturbed and unwell when they are on observations than when they are not, and it is unsurprising that individuals engage in more adverse incidents when they are more labile and agitated than when they are stable and settled. What is not known from these data is what specific effect CSO had on adverse incidents; it is probable that had those patients not been on constant observations, the level of adverse incidents in such an unsettled group would have been much higher. One motivation for CSO is to manage risk with patients at risk of harming themselves or others, but assessing the extent to which CSO is successful in doing that can be problematic. One explanation of perpetrators' decisions to engage in aggression involves a calculation of the effect/danger ratio,^11^ where an individual judges the costs and benefits of using aggression in any given situation, opting to use aggression only when the costs to them in terms of detection and reprimand are limited relative to the potential outcome. As such, less serious forms of challenging behaviour can occur as a result of this cost–benefit analysis, and, for individuals unable to manage their use of challenging behaviour, being on observations could encourage the behaviour least likely to elicit reprimand (e.g. more minor incidents such as verbal abuse and property damage). This may explain why the increase in incidents while on CSO was largely due to an increase in category D incidents, and a rational assumption is that the effect of constant observations may have been to deflect what was a potentially serious situation into a more minor incident.

Across the different units, specific concerns about aggression and self-harm were the reasons for initiating most of the CSO hours, but the balance between these reasons was markedly different in different settings. Aggression was most frequently the reason for constant observations in the male medium and low secure settings, but self-harm was the most prevalent reason for constant observations in the female MSU ward and in the HSS. In fact, nearly all instances of CSO on the female ward were motivated by actual, or risk of, self-harm. The dominance of self-harm as a driver of CSO in this setting may be partly related to diagnosis. The present data-set did not drill down into the specific diagnoses of patients in each unit, but a recent large-scale survey of forensic psychiatric in-patients in The Netherlands^12^ found that, while around 75% of both male and female patients were diagnosed with both axis I and axis II disorders, 61% of female patients had a diagnosis of borderline personality disorder (BPD) and a further 21% presented with borderline traits. BPD diagnoses were much less prevalent in the male patients in their sample. A separate and equally valid explanation relates to clinician perception and response. There is recognition in secure psychiatric services that clinicians' responses to aggression frequently differ depending on whether the patient is male or female; aggression by men leads to more discussion by clinicians than aggression by women, and aggression generally is viewed as a male phenomenon in spite of the evidence that women may be equally aggressive.^13^^,^^14^ Similarly, the prevalent clinical view is that self-harm is more common in women than men, in spite of more equivocal evidence in the literature.^15^^,^^16^ Further exploration of this issue falls outside the scope of the current research, but the present data may support the view that a potential gender bias exists in clinical responses to challenging behaviours.

Although findings from these data should be viewed in the context of a single mental health trust (and a single female ward), they nevertheless present a picture of the prevalence and use of a cornerstone of clinical practice in mental healthcare.

## About the authors

**Katie Lambert**, Research Assistant, Mersey Care NHS Foundation Trust; **Simon Chu**, Senior Lecturer in Psychology, University of Central Lancashire and Research Fellow, Ashworth Research Centre, Mersey Care NHS Foundation Trust; **Chris Duffy**, Assistant Psychologist, **Victoria Hartley**, Research Assistant, and **Alison Baker**, Senior Clinical Nurse, all at Mersey Care NHS Foundation Trust; **Jane L. Ireland**, Professor of Forensic Psychology, University of Central Lancashire and Consultant Forensic Psychologist, Mersey Care NHS Foundation Trust.
